# Concordances and differences between a unidimensional and multidimensional assessment of frailty: a cross-sectional study

**DOI:** 10.1186/s12877-019-1369-7

**Published:** 2019-12-10

**Authors:** Michael C. J. Van der Elst, Birgitte Schoenmakers, Linda P. M. Op het Veld, Ellen E. De Roeck, Anne Van der Vorst, Gertrudis I. J. M. Kempen, Nico De Witte, Jan De Lepeleire, Jos M. G. A. Schols, And D-SCOPE Consortium, A. S. Smetcoren, S. Dury, L. De Donder, E. Dierckx, D. Lambotte, B. Fret, D. Duppen, M. Kardol, D. Verté, L. J. Hoeyberghs, N. De Witte, E. E. De Roeck, S. Engelborghs, P. P. De Deyn, M. Van der Elst, J. De Lepeleire, B. Schoenmakers, A. van der Vorst, G. A. R. Zijlstra, G. I. J. M. Kempen, J. M. G. A. Schols

**Affiliations:** 1 Department of Public Health and Primary Care, KU Leuven, Kapucijnenvoer 33 bus 7001, B-3000 Leuven, Belgium; 2 Department of Health Services Research, Care and Public Health Research Institute (CAPHRI), Maastricht University, Maastricht, the Netherlands; 3 Centre of Research Autonomy and Participation for Persons with a Chronic Illness, Faculty of Health, Zuyd University of Applied Sciences, Heerlen, the Netherlands; 4 Department of Clinical and Lifespan Psychology, Vrije Universiteit Brussel, Brussels, Belgium; 5 Laboratory of Neurochemistry and Behavior, University of Antwerp, Antwerp, Belgium; 6 Faculty of Psychology and Educational Sciences, Vrije Universiteit Brussel, Brussels, Belgium; 7 Faculty of Education, Health and Social Work, University College Ghent, Ghent, Belgium; 8 Department of Health Services Research, Family Medicine, Faculty of Health, Medicine and Life Sciences, Maastricht University, Maastricht, the Netherlands

**Keywords:** Frailty, Frailty measurements, Frailty Phenotype, Multidimensional frailty, Older adults

## Abstract

**Background:**

Many instruments to identify frail older people have been developed. One of the consequences is that the prevalence rates of frailty vary widely dependent on the instrument selected. The aims of this study were 1) to examine the concordances and differences between a unidimensional and multidimensional assessment of frailty, 2) to assess to what extent the characteristics of a ‘frail sample’ differ depending on the selected frailty measurement because ‘being frail’ is used in many studies as an inclusion criterion.

**Method:**

A cross-sectional study was conducted among 196 community-dwelling older adults (≥60 years), which were selected from the census records. Unidimensional frailty was operationalized according to the Fried Phenotype (FP) and multidimensional frailty was measured with the Comprehensive Frailty Assessment Instrument (CFAI). The concordances and differences were examined by prevalence, correlations, observed agreement and Kappa values. Differences between sample characteristics (e.g., age, physical activity, life satisfaction) were investigated with ANOVA and Kruskall-Wallis test.

**Results:**

The mean age was 72.74 (SD 8.04) and 48.98% was male. According to the FP 23.59% was not-frail, 56.92% pre-frail and 19.49% frail. According to the CFAI, 44.33% was no-to-low frail, 37.63% was mild frail and 18.04% was high frail. The correlation between FP and the CFAI was r = 0.46 and the observed agreement was 52.85%. The Kappa value was κ = 0.35 (quadratic κ = 0.45). In total, 11.92% of the participants were frail according to both measurements, 7.77% was solely frail according to the FP and 6.21% was solely frail according to the CFAI. The ‘frail sample respondents’ according to the FP had higher levels of life satisfaction and net income, but performed less physical activities in comparison to high frail people according to the CFAI.

**Conclusion:**

The present study shows that the FP and CFAI partly measure the same ‘frailty-construct’, although differences were found for instance in the prevalence of frailty and the composition of the ‘frail participants’. Since ‘being frail’ is an inclusion criterion in many studies, researchers must be aware that the choice of the frailty measurement has an impact on both the estimates of frailty prevalence and the characteristics of the selected sample.

## Background

Frailty is an emerging concept, although no agreement exists about its definition [1, 2]. Consequently, many instruments for identifying frail older adults have been developed [3]. Initially, frailty was often designated as a unidimensional construct, defined as “a medical syndrome with multiple causes and contributors that is characterized by diminished strength, endurance, and reduced physiologic function that increases an individual’s vulnerability for developing increased dependency and/or death” [4]. An example of such a (bio)medical, unidimensional approach to operationalize frailty is the Fried Phenotype (FP) [5]. Nowadays, some conceptual models of frailty attempt to be integrative [6]. Such an integrative approach has a multidimensional perspective, and in addition to physical features such as grip strength or endurance, social and/or psychological domains are also included [7, 8]. More recently, the environmental domain has been added as well [9]. An example of an integrative, multidimensional definition of frailty is: “A dynamic state affecting an individual who experiences losses in one or more domains of human functioning (physical, psychological, social), which is caused by the influence of a range of variables and which increases the risk of adverse outcomes” [2]. In line with this, the Comprehensive Frailty Assessment Instrument (CFAI) for instance, is an assessment with a multidimensional perspective on frailty [9].

Prior research showed that the different operationalizations of frailty have an important impact on the classification of older adults as frail or not-frail, as a consequence the prevalence of frailty differ widely across studies depending on the used frailty assessment [10]. According to a systematic review, the prevalence estimates of frailty range from 4.0% till 59.1% [10]. In addition, a previous study, comparing four frailty scales in the same population estimated a prevalence rate of frailty ranging between 22.2% (FP) and 64.8% (Tilburg Frailty Indicator, TFI) [11]. Furthermore, Ntanasi et al. compared five frailty scales whereby the prevalence ranged from 4.1% until 30.2%, but less than 1% was frail according to all scales. Depending on the used frailty scale the characteristics of the ‘frail sample’ had important differences. For instance, 50% of the ‘frail sample’ according to the FP was 80 years and over, while this was only 20.1% of the frail older persons as assessed according to the Groningen Frailty Index (GFI) [12]. A study of Aguayo and colleagues comparing 35 frailty measurements found considerably varying prevalence rates across studies (ranged 1.6% for men till 72.4% for women) [13].

Therefore, one can assume that these differences between frailty measurements also could have a strong influence on the outcomes of a study. Since ‘being frail’ is often used as an inclusion criterion, the selected frailty measurement may have a major impact on how many older adults will be included. Furthermore, one can expect that depending on the selected frailty assessment differences occur on who will be included and also with respect to the characteristics of the selected frail sample (e.g., differences in the average age of the sample). In the literature, many studies can be found which compared the predictive validity of different frailty-instruments or the risk factors between frailty-instruments [11, 12, 14]. However, the impact of the used frailty measurements on the characteristics of a selected ‘frail sample’ is not yet assessed and remains unclear.

The aim of this study was to examine the concordances and differences between a unidimensional (FP) and a multidimensional assessment (CFAI) of frailty and to assess to what extent the characteristics of ‘frail participants’ differ depending on the used frailty measurement. Since the FP solely focuses on the physical domain, while the CFAI adds measures within the psychological, social and environmental domain as well, one can assume that particularly some agreement will be found because of the mutual physical domain and that differences will be found because of the additional domains.

## Method

### Study design

Data were gathered within the D-SCOPE project (Randomized Controlled Trial), which stands for Detection, Support and Care for Older people: Prevention and Empowerment. The aim of D-SCOPE was to detect frail community-dwelling older adults who previously were unnoticed and to improve their access to tailored care and support [15]. Participants were selected from the census records of three municipalities in Flanders, the northern region of Belgium (Ghent, Knokke-Heist and Thienen) and were all community-dwelling older adults and aged ≥60 years. Participants were excluded from the study in case of hospitalization, when the participant or the informal caregiver indicated that the older adult was unable to participate, or when the interviewer noted that the older participant was unable to provide adequate answers (e.g., not being able to answer questions due to physical exhaustion or decreasing attention). To determine the numbers of participants needed for the present cross-sectional study, a sample size calculation was conducted (see Additional file 1: Figure S1. Sample size). Therefore, only a part of the D-SCOPE participants were asked to do the performance-based tests (Walk time and Handgrip strength) and were included in the present study [16]. Data collection was retrieved by two assessors (authors MVDE and AvdV) and started in March 2017 and lasted until September 2017. The details of the data collection method of D-SCOPE can be found elsewhere [15]. This study was reviewed and approved by the medical ethics committee of the Vrije Universiteit Brussel, Brussels, Belgium (reference number: B.U.N. 143,201,630,458). Written consent was obtained from all participants. The study adheres to the STROBE guidelines.

#### Frailty measurements

Fried’s Phenotype of frailty was used to measure unidimensional frailty. According to the Fried Phenotype the following five criteria are used to determine the level of frailty: weight loss, exhaustion, low physical activity, slowness, and weakness [5]. Weight loss is measured by asking the following question: “In the past year, have you lost more than 5 kg unintentionally (i.e., not due to dieting or exercise)?” If yes, the participant was scored frail for the weight loss criterion. Exhaustion was determined using two questions of the CES–D Depression Scale, for which the following two statements had to be answered on a scale from 0 to 3: “Last week, I felt that everything I did was an effort”; and “Last week, I could not get going”. Participants could answer with the options: 0 = rarely or none of the time (< 1 day), 1 = some or a little of the time (1–2 days), 2 = a moderate amount of the time (3–4 days), or 3 = most of the time. The participants answering “2” or “3” on at least one of these two questions were categorized as frail by the exhaustion criterion [17]. Low physical activity was measured by asking the participants whether they did any physical activities (e.g., walking, swimming, or cycling). The answer options were never, rarely, monthly or weekly [18]. Participants answering weekly were categorized as not-frail, the others as frail. For the performance-based measures slowness and weakness, all participants received standardized instructions. For the slowness criterion, participants were asked to walk 4.57 m (15 ft.) at a normal pace, starting from a standing position. Equal to the original criteria from Fried and colleagues, depending on gender and height, a walk time below 6 or 7 s was categorized as not-frail, the others were considered as frail [5, 17]. Weakness (handgrip strength) was measured using a Saehan hand dynamometer (Saehan Corporation, South Korea). Participants were asked to squeeze the dynamometer as hard as possible. Depending on gender and BMI, a different cut-off existed to categorize a person as (not-)frail [5]. In Additional file 2: Text S1, the protocol of the performance-based tests is described in detail. The result of each frailty criterion is dichotomized: frail (score 1) or not-frail (score 0). The final frailty sum scores range from 0 to 5. A score of 0 means a not-frail participant, participants with a score of 1 or 2 are considered pre-frail, and a score of 3–5 indicates that someone is frail [5].

The Comprehensive Frailty Assessment Instrument (CFAI) is self-report and was used to measure multidimensional frailty. This frailty measurement includes four domains: physical, social, psychological, and environmental [9]. The physical domain (4 items) assesses an older adult’s functionality. An example of an item is ‘Walking up a hill or some stairs’. The psychological domain (8 items) measures mood-disorders and emotional loneliness such as ‘I feel unhappy and depressed’. The social domain (4 items) assesses social loneliness and the potential social support network like ‘There are enough people whom I can rely on when I am in trouble’. Finally, the environmental domain (5 items) evaluates the suitability of the physical housing environment, for instance, ‘My house is in a bad condition/poorly kept’. All subscales range theoretically from 0 to 100 with higher scores indicating more severe levels of frailty. An overall score on the CFAI is calculated by summing the scores on each domain divided by the number of domains. A detailed description of the CFAI can be found in Additional file 3: Text S2. The composition of the CFAI makes it possible to analyze the overall scale, including all the domains, but also on the domains separately. In what follows, CFAI is always used to indicate the overall scale including all the domains; otherwise, the specific domain is mentioned. A previous study determined the presence of three natural groups for the CFAI and its four domains: no-to-low frail, mild frail and high frail. The cut-offs of the CFAI are 0–21.89, 21.90–38.79 and 38.80–100, respectively (Additional file 3: Text S2, Table C) [19].

#### Characteristics of participants

Participants were asked their date of birth and net monthly income. Meaning in life was evaluated with a short version of the Meaning in Life Questionnaire [20]. Life satisfaction was measured by using the Satisfaction with Life Scale, a validated scale which measures global life satisfaction [21]. To assess mastery, a questionnaire with 4 items was used which evaluates to what extent people feel they exert control over existing circumstances of their lives [22]. In addition, 1 self-constructed item assesses mastery in relation to others [23]. Social inclusion was measured by using 1 item from the Community Integration Measure: to what extent they feel like part of the community [24]. Ageing well in place was assessed using a self-constructed question: to what extent the older participant feels he/she lives at home in a qualitative way. Feeling frail was assessed by 1 item: to what extent the older participant feels frail [15].

### Statistical analyses

To describe the sample, univariate analyses were conducted. To assess concordances and differences between the FP and the CFAI several tests were applied. First, differences in mean scores for the CFAI and the four CFAI-domains according to the three levels of the FP were examined by means of Analysis of Variance (ANOVA). As post-hoc, the Tukey HSD test was conducted to find differences in mean between pairs. Second, the strength of the association between the FP and both the CFAI and its subdomains was assessed by calculating Spearman correlation coefficients. Stronger correlations indicate concordance between the concepts that are measured, lower correlations indicate differences. No definite cut-offs exist, however Reid et al. suggested that different tests of the same construct should have correlation coefficients greater than 0.30, therefore, > 0.30 was used as the cut-off [25]. Third, the observed agreement and the Kappa value (interrater reliability) between the FP and the CFAI (and domains) were computed [26]. Since both frailty scales are ordinal, a weighted (Linear and Quadratic) Kappa Value was analyzed. The interpretation of the weighted Kappa value was divided as follows: ≤0, no agreement; 0.01 to 0.20, none to slight; 0.21 to 0.40, fair; 0.41 to 0.60, moderate; 0.61 to 0.80, substantial; 0.81 to 1.00, almost perfect [27]. Since ‘being frail’ is an inclusion criterion in many studies, it was decided to compare the characteristics (as mentioned above) of the frail sample according to the FP with the frail sample of the CFAI. ANOVA was conducted for continuous variables and the Kruskall-Wallis tests for ordinal variables. Missing data were excluded pairwise. Statistical significance was set at *p* < 0.05 for all analyses, which were performed using SPSS 24 (IBM SPSS Statistics for Windows, IBM Corp., Armonk, NY).

## Results

In total, 196 people aged 60 years or older participated in the study. The mean age was 72.74 years old (SD 8.04, range 60–93) and 48.98% was male. The characteristics of the sample are described in Table 1. According to the FP measurement, 19.49% of the participants were frail, while 18.04% of the participants were high frail according to the CFAI. According to the CFAI 44.33% of the sample was no-to-low frail, while 23.59% was not-frail according to FP.

**Table 1:** Socio-demographic characteristics of the sample (*N* = 196)

	Mean (SD)	N (%)
Age	72.74 (8.04)	
Gender		
Male		96 (48.98)
Female		100 (51.02)
Marital status		
Married		61 (31.12)
Never married		14 (7.14)
Divorced		42 (21.43)
Cohabited		26 (13.27)
Widow(ed)		53 (27.04)
Highest level of education		
No/primary		8 (4.10)
Lower secondary		58 (29.74)
Higher secondary		77 (39.49)
Higher education		52 (26.67)
Relocated past 10 years		
Yes		97 (49.49)
No		99 (50.51)
Origine (country of birth)		
Belgium		176 (89.80)
Other		20 (10.20)
Net Income in Euro’s		
500–999		10 (6.13)
1000–1499		63 (38.65)
1500–1999		32 (19.63)
2000+		58 (35.58)
Fried Phenotype (unidimensional)		
Not-frail		46 (23.59)
Pre-frail		111 (56.92)
Frail		38 (19.49)
CFAI^a^ (multidimensional)		
No-low frail		86 (44.33)
Mild frail		73 (37.63)
High frail		35 (18.04)

^a^*CFAI* Comprehensive Frailty Assessment Instrument

Table 2 shows that frail participants according to FP, scored significantly higher on the CFAI and the CFAI-domains physical and psychological frailty compared to people who were not-frail or pre-frail. No such differences were found for the CFAI-domains environmental and social frailty.

**Table 2:** The CFAI mean scores (total and per domain) according to the Fried Phenotype distribution

	N	Not-frail (mean ± SD)	Fried Phenotype	Frail (mean ± SD)	*P*-value
			Pre-frail (mean ± SD)		
CFAI	193	19.35 ± 10.88^1^	23.84 ± 11.61^2^	41.05 ± 14.28^12^	*p* < 0.000
CFAI Physical	195	6.25 ± 13.11^34^	22.07 ± 30.61^35^	61.84 ± 32.22^45^	*p* < 0.000
CFAI Psychological	193	14.96 ± 16.32^6^	16.46 ± 15.52^7^	36.95 ± 22.92^67^	*p* < 0.000
CFAI Social	195	46.61 ± 18.70	45.38 ± 18.04	51.21 ± 20.48	ns
CFAI Environmental	195	9.57 ± 13.16	10.99 ± 12.23	14.21 ± 14.64	ns

Anova test. According to the Levene’s Statistic, the variance of CFAI Physical and CFAI psychological were not equal instead the Welch test and Brown-Forsythe test used to determine the *p*-value. As post-hoc, the Tukey HSD test was conducted to find differences in mean between pairs (see the superscripts). Superscripts with the same number indicate a significant mean difference between two pairs of groups. The CFAI and its domains are a continuous scale (0–100). For the psychological domain, there were missing data for two participants and, for the Fried Phenotype one participant had missing data. ns = non-significant

Table 3 presents the results of the Spearman correlation analysis, observed agreement and kappa value. The Spearman correlation between the FP and the CFAI was R = 0.46, which was mainly attributed to by the physical domain (R = 0.52), and to a lesser extent by the psychological domain (R = 0.34). The correlations between the FP and the social and environmental domain were R = 0.05 and R = 0.13, respectively. The observed agreement between the FP and the CFAI was 52.85%, the Kappa value was linear weighted = 0.35 and quadratic weighted = 0.45. Additional file 4: Table S1 presents the number of participants for the different levels of frailty according to the FP and the CFAI and its domains.

**Table 3:** Measurements for differences and concordances between the Fried Phenotype and the CFAI and its domains

	Fried Phenotype			
	Spearman correlation	Observed agreement	Weighted Kappa value	Quadratic weighted Kappa value
CFAI	R = 0.46	52.85%	0.35	0.45
CFAI Physical	R = 0.52	44.62%	0.25	0.39
CFAI Psychological	R = 0.32	39.38%	0.18	0.28
CFAI Social	R = 0.05	40.05%	0.04	0.06
CFAI Environmental	R = 0.13	48.72%	0.13	0.14

Spearman correlation: The same construct should have correlation coefficients greater than 0.30. The interpretation of the weighted Kappa coefficient is divided as follows: < 0, no agreement; 0.01 to 0.20, none to slight; 0.21 to 0.40, fair; 0.41 to 0.60, moderate; 0.61 to 0.80, substantial; 0.81 to 1.00, almost perfect

In total, 23 participants (11.92%) were frail according to both the FP and the CFAI, 15 participants (7.77%) were solely frail according to FP, and 12 participants (6.21%) were solely high frail according to the CFAI (see Additional file 4: Table S1, Table F for the subdomains of the CFAI).

The characteristics of the frail samples differed, depending on the used frailty measurement (Table 4). For instance, life satisfaction was significantly lower in the respondents who were high frail according to the CFAI compared to people who were frail according to the FP. The high frail sample according to the CFAI tended to have a lower income in comparing with the frail sample of the FP; with regard to physical activities, the frail FP-sample tended to do less physical activities than the high frail sample according to the CFAI. For the characteristics age, meaning in life, social inclusion and feeling frail no significant differences between groups were found.

**Table 4:** Characteristics of the frail samples according to the frailty measurements (CFAI and Fried Phenotype)

			Solely CFAI High frail	CFAI and FP (High-) frail	Solely FP Frail	*p*-value
			*N* = 12	*N* = 23	*N* = 15	
Age	mean		70.00	75.04	76.67	0.141
Sense of Mastery (0–5)	mean		3.36	2.94^1^	3.76^1^	0.003*
Meaning in Life (0–5)	mean		3.67	3.53	3.87	0.427
Life Satisfaction (0–5)	mean		2.82^2^	3.07^3^	3.92^23^	0.001*
Social Inclusion (0–5)	mean		3.58	3.87	4.38	0.199
Ageing Well in Place (0–5)	mean		4.17	3.74^4^	4.53^4^	0.081¥
Feeling Frail (0–5)	mean		3.25	3.17	2.73	0.465
Net income in Euro’s	N					0.021
		500–999	2	2	0	
		1000–1499	9	10	4	
		1500–1999	0	4	5	
		2000 or more	1	5	4	
Physical activities	N					0.001
		never	2	18	11	
		Rarely	1	2	0	
		monthly	1	1	0	
		weekly	8	2	4	

Continuous variables were tested by ANOVA (Post hoc: Tukey HSD), while ordinal variables were tested by the Kruskall-Wallis test (post hoc Kendall tau). CFAI=Comprehensive Frailty Assessment Index, FP = Fried Phenotype. *p* < 0.05 is considered significant. *p* < 0.10 is considered a trend. Superscripts with the same number indicate a significant (mean) difference between two pairs of groups. Net income and physical activity are significant different between solely CFAI and the two other groups (solely FP/CFAI and FP). Except age, all continuous scales ranged from 0 indicating a low level of … (e.g., mastery), till 5 indicating a high level of … (e.g., mastery)

## Discussion

This study aimed to examine the concordances and differences between a unidimensional (FP) and a multidimensional assessment (CFAI) of frailty and to assess to what extent the characteristics of ‘frail participants’ differ depending on the used frailty measurement. The results show that FP and CFAI measure partly the same ‘frailty-construct’, with a fair (linear weighted Kappa) to moderate (quadratic weighted Kappa) resemblance. Both scales (FP and CFAI) indicate that 18 to 19% of the participants belong to the highest level of frailty. Although, the frailest group in both scales (FP and CFAI) overlaps only partially for instance, 7.77% was solely frail according to FP and not according to the CFAI. Besides differences in ‘frailty status’ between the samples, the present results also shows some differences between the characteristics of these ‘frail samples’.

With regard to the first aim, the results show that the overlap between CFAI and FP seems mainly due to the physical domain of the CFAI and to a lesser extent the psychological domain. Because the FP includes several physical elements, like physical activity, walk time and handgrip strength, the relation with the CFAI’s physical domain was expected [5]. In addition, an association with the psychological domain of the CFAI could have been expected since exhaustion is also seen as a characteristic of depressive symptoms [28].

Differences between both frailty measurements were found. For instance, more participants were categorized as no-to-low frail according to the CFAI compared to the FP. In addition, some participants were frail according to FP and not according the CFAI and vice-versa. This confirms previous research. For instance a prior study of Ntanasi et al. (2018) examined the overlap of ‘frail participants’ using 5 frailty scales; the results show only a small overlap (0.7%), while some instruments indicated a frailty prevalence of 30.2% [12]. Further, low correlations were found between the FP and the social and environmental domain of the CFAI. Since both domains are not included in the FP, a weak or no association was expected. The inclusion of extra domains in the CFAI is probably also the reason why the Kappa value between the FP and the CFAI was only fair (linear weighted) to moderate (quadratic weighted). However, also between the physical domain of the CFAI and the FP differences were found; the interrater reliability was here only fair. This difference can be due to the use of performance-based tests (FP) versus self-reporting questions (CFAI) [29–31].

Concerning the characteristics of the ‘frail samples’, it was shown that depending on the used frailty measurement the characteristics of the study samples differed. Since the FP is focusing on physical frailty, one could expect that the frail sample according to the FP would be physically weaker in comparison with the frail sample of the CFAI, since the other domains in the CFAI will downsize the importance of the physical domain. This can explain why frail participants according to the FP were older and physically less active. Although, it must be pointed out that physical activity is one of the criteria of the FP (see Additional file 5: Table S2: physical activity). The frail CFAI-sample seems to have lower levels of life satisfaction and social inclusion. Furthermore, the frail CFAI-sample also seems to have a lower income. This is consistent with previous research that found that income was a risk factor for multidimensional frailty and not for unidimensional frailty (as measured with the FP) [12].

### Strengths and limitations

A strength of the present study is the difference in focus in comparison with other studies. Whereby previous research often aims to assess the predictive validity of different frailty-instruments or to find similarities in frailty-instruments, the present study objective is to find concordances and differences between two frailty-instruments [11, 12]. Thereby the one frailty scale is not considered to be better or worse as the other, but both can have an added value depending on the context.

This study has some limitations as well. First, the criterion low physical activity was not operationalized in exactly the way it was initially proposed in the FP; this may have affected the results [5]. Secondly, the sample to assess to what extent the characteristics of ‘frail participants’ differ depending on the used frailty measurement, was rather small. Thirdly, only a small set of characteristics were assessed, and variables such as multi-morbidity, total number of drugs used, were not available [32].

### Implications and future research

Many frailty measurements exist, each with their specific qualifications. Consequently, differences occur between frailty measurements, for instance in the classification of older adults and the prevalence of frailty. Since the choice of a specific frailty measurement has an impact on the selected sample and the characteristics of the sample, we assume that the outcomes of a (intervention) study can differ as well depending on the used frailty measurement. We also assume that both approaches of frailty examined in the present study can be useful for distinct purposes, or contexts. For instance, an intervention study focusing on preventing the incidence of falls or the improvement of physical activity will probably make the best use of an approach where particularly physical elements of frailty are included. The selected sample will be physically weaker (e.g., presence of sarcopenia, doing less physical activities) when a physical unidimensional approach is used compared to a multidimensional approach of frailty whereby social, psychological and environmental domains are included and may downsize the importance of the physical domain. In case of an intervention study focusing on ageing (well) in place, one can assume that a multidimensional approach of frailty will probably have a higher likelihood to recruit the targeted sample population. Since ageing well in place is partly determined by the social network of older adults [33], a multidimensional approach of frailty including a social domain will probably be better able to recruit those older adults at risk for institutionalization, for example because of a lacking social network; this group may be less recruited in a unidimensional physical approach of frailty [34]. To achieve a maximum effect of an intervention, we assume that the ability to recruit the targeted sample is essential. Although more research is needed to find evidence that both approaches of frailty can be useful for distinct purposes, or contexts.

Researchers must be aware that different methods to operationalize and measure frailty may have important consequences for the outcomes of a study. Therefore, more research including both a unidimensional and a multidimensional approach in larger samples is warranted. A better understanding of the similarities and differences in frailty approaches and their consequences for the effectiveness of interventions and which approach is recommendable for which purpose will offer healthcare professionals a better framework, which they can apply to improve the care for frail older adults.

## Conclusion

The present study shows that the FP and CFAI partly measure the same construct, although differences were found in the prevalence of frailty and the composition of the ‘frail participants’. Since ‘being frail’ is an inclusion criterion in many studies, researchers must be aware that the choice of the frailty measurement has an impact on both the estimates of frailty prevalence and the characteristics of the selected sample.

## Additional Files

**Additional file 1: Figure S1.** Sample size.

**Additional file 2: Text S1.** Protocol performance-based tests Fried Phenotype.

**Additional file 3: Text S2.** The Comprehensive Frailty Assessment Instrument (CFAI).

**Additional file 4: Table S1.** Observed agreement between Fried Phenotype and CFAI.

**Additional file 5: Table S2.** Physical activity.

## Data Availability

The datasets generated and/or analysed during the current study are not publicly available due the ethics approval for this study not allowing open access to the individual participant data but are available from the corresponding author on reasonable request.

## Abbreviations

CES-D: Center for Epidemiological Studies Depression Scale
CFAI: Comprehensive Frailty Assessment Instrument
D-SCOPE: Detection, Support and Care for Older people: Prevention and Empowerment
FP: Fried Phenotype
GFI: Groningen Frailty Index
RCT: Randomised controlled trial
TFI: Tilburg Frailty Indicator
