# Supplementary material for: Concordances and differences between a unidimensional and multidimensional assessment of frailty: a cross-sectional study
Source: BMC Geriatr. 2019 Dec 10;19:346. doi: 10.1186/s12877-019-1369-7 (PMC6902576; doi:10.1186/s12877-019-1369-7)
Supplement: Supplementary file 1 — Additional file 1: Figure S1. Sample size. [file 12877_2019_1369_MOESM1_ESM.docx]

**Additional Figure S1: Sample size**
