# Supplementary material for: Concordances and differences between a unidimensional and multidimensional assessment of frailty: a cross-sectional study
Source: BMC Geriatr. 2019 Dec 10;19:346. doi: 10.1186/s12877-019-1369-7 (PMC6902576; doi:10.1186/s12877-019-1369-7)
Supplement: Supplementary file 2 — Additional file 2: Text S1. Protocol performance-based tests Fried Phenotype. [file 12877_2019_1369_MOESM2_ESM.docx]

**Additional Text S1: Protocol performance-based tests Fried Phenotype**

- Weight loss was measured by asking: In the last year, have you lost more than 10 pounds unintentionally (i.e., not due to dieting or exercise)? If yes, then the participant was scored frail for weight loss criterion.
- Exhaustion was determined using the CES–D Depression Scale, for which the following two statements were read by one of the two assessors: (a) last week, I felt that everything I did was an effort; and (b) last week, I could not get going. Participants could answer with the options: 0 = rarely or none of the time (<1 day), 1 = some or a little of the time (1–2 days), 2 = a moderate amount of the time (3–4 days), or 3 = most of the time. The participants answering “2” or “3” on at least one of these two questions are categorized as frail by the exhaustion criterion.
- Low physical activity was measured by asking the participants whether they did any sports activities (e.g., walking, swimming, or cycling). The answer options were never, rarely, monthly or weekly. Participants answering weekly were categorized as not-frail, the others as frail.

For both performance-based measures, all participants received standardized Instructions.

- Slowness, participants were asked to walk 4.57 m (15 ft) at a normal pace, starting from a standing position. No encouragement was given by the assessor. A walking aid was permitted if necessary. The test was performed three times, and each time the time they needed was measured. For analyses, the average time was used. To indicate the distance, a rope was used.
- Weakness (handgrip strength) was measured using a Saehan hand dynamometer (Saehan Corporation, South Korea). Participants were asked to press as hard as possible on the dynamometer. Three measurements per hand were conducted alternately with a minimum of 30 seconds rest between each attempt. Results were averaged per hand, and the highest average score (e.g., either left or right hand, it did not matter whether this was their ‘dominant hand’), was used for analyses. Before doing the handgrip strength test, participants were asked to practice first. Participants were seated upright in a chair without armrests or standing, the shoulder and forearm in neutral position, and the elbow in 90° flexion. The handle position of the dynamometer was determined in such a way that the intermediate phalanges were on the front side of the handle. Participants were verbally encouraged.

For the cut-off values of the performance-based Fried measures, it is necessary to measure weight and height, in order to calculate the Body Mass Index (BMI). Cut-off values for both performance-based measures were used as described by Fried and colleagues (5). To measure the weight of the participants both researchers used a scale of the brand OMRON, participants were weighted twice. The average body weight was calculated and used in the analysis. To measure body length, participants were asked to stand against a wall as straight as possible (without shoes). A book with a hard cover was placed on the respondent’s head and a post it was used to mark the respondent’s body length. Using a ruler, the body length was measured. This procedure was done three times, and the average body length was used in the analysis. The assessors were trained to conduct the data collection by author LOhV.

*Cut-offs performance-based tests Fried Phenotype*

- Walk Time, stratified by gender and height (gender-specific cutoff a medium height).

Men

Height ≤ 173 cm 7 seconds

Height > 173 cm 6 seconds

Women

Height ≤ 159 cm 7 seconds

Height > 159 cm 6 seconds

- Grip Strength, stratified by gender and body mass index (BMI) quartiles:

Men

BMI ≤ 24 ≤ 29

BMI 24.1–26 ≤ 30

BMI 26.1–28 ≤ 30

BMI > 28 ≤ 32

Women

BMI ≤ 23 ≤ 17

BMI 23.1–26 ≤ 17.3

BMI 26.1–29 ≤ 18

BMI > 29 ≤ 21
