# Supplementary material for: Concordances and differences between a unidimensional and multidimensional assessment of frailty: a cross-sectional study
Source: BMC Geriatr. 2019 Dec 10;19:346. doi: 10.1186/s12877-019-1369-7 (PMC6902576; doi:10.1186/s12877-019-1369-7)
Supplement: Supplementary file 3 — Additional file 3: Text S2. The Comprehensive Frailty Assessment Instrument (CFAI). [file 12877_2019_1369_MOESM3_ESM.docx]

**Additional Text S2: The Comprehensive Frailty Assessment Instrument (CFAI)**

1. Have the following activities been hampered by your state of health? If so, for how long? (Please tick all appropriate items)

|  | Not at all | 3 months or less | More than 3 months |
| --- | --- | --- | --- |
| Less demanding activities like carrying shopping bags |  |  |  |
| Walking up a hill or some stairs |  |  |  |
| Bending or lifting |  |  |  |
| Going for a walk |  |  |  |

2. Considering the last few weeks, to which extent do you agree with the following (please tick)

1 = not at all 3 = more than usual

2 = not more than usual 4 = considerably more than usual

|  | 1 | 2 | 3 | 4 |
| --- | --- | --- | --- | --- |
| I feel unhappy and depressed |  |  |  |  |
| I feel like I’m losing my self-confidence |  |  |  |  |
| I feel like I cannot cope with problems |  |  |  |  |
| I feel like I’m under constant pressure |  |  |  |  |
| I feel like I’m not worth anything anymore |  |  |  |  |

3. To which extent do you agree with the following statements? (please tick all appropriate items)

1 = I completely disagree 4 = I agree

2 = I disagree 5 = I completely agree

3 = I neither agree nor disagree

|  | 1 | 2 | 3 | 4 | 5 |
| --- | --- | --- | --- | --- | --- |
| I experience a general sense of emptiness |  |  |  |  |  |
| I miss having people around me |  |  |  |  |  |
| I often feel rejected |  |  |  |  |  |
| There are enough people whom I can rely on when I am in trouble |  |  |  |  |  |
| I know many people whom I can totally trust |  |  |  |  |  |
| There are enough people with whom I feel a bond |  |  |  |  |  |
| My house is in a bad condition/poorly kept |  |  |  |  |  |
| My house is not very comfortable |  |  |  |  |  |
| It is difficult to heat my house |  |  |  |  |  |
| There is insufficient comfort in my house |  |  |  |  |  |
| I do not like my neighborhood |  |  |  |  |  |

4. Suppose you are unable to carry out the activities you usually do in terms of housekeeping for a certain length of time; who would you be able to appeal to? (Please tick all appropriate items) (More than one answer may be given)

| Partner |  |
| --- | --- |
| Son |  |
| Daughter-in-law |  |
| Daughter |  |
| Son-in-law |  |
| Grandchild |  |
| Sister or brother (in-law) |  |
| Family |  |
| Neighbours |  |
| Friends |  |

*Calculation of the scores of the CFAI:*

The purpose of the CFAI was not only to assess 4 domains of frailty, but also to give equal weight to the four domains. Table A gives an overview of those domains, their measurements, their scores, their weight within the domain (WWD) and the weight of the domain within the total score of the CFAI (DWWT). In Table B the formulas for calculating the domain and total scores that are presented in Table A (18) .

Table A CFAI-domains, measurements and weights

| **CFAI** | **DWWT** | **Measurements** | **Min-max** | **WWD** |
| --- | --- | --- | --- | --- |
| CFAI Physical domain | 25% | Physical items | 0-8 | 100% |
| CFAI Psychological domain | 25% |  |  |  |
|  |  | Mood disorders | 0-15 | 50% |
|  |  | Emotional loneliness | 0-12 | 50% |
| CFAI Social domain | 25% |  |  |  |
|  |  | Social loneliness | 0-12 | 50% |
|  |  | Social support network | 0-10 | 50% |
| CFAI Environmental domain | 25% | Actual housing/environment | 0-20 | 100% |

Table B Formulas for calculating the subdomains of the CFAI

| **CFAI** | **Formula** |
| --- | --- |
| CFAI Physical domain | [Physical items]*100/8 |
| CFAI Psychological domain | [mood disorders]*50/15 + [emotional loneliness]*50/12 |
| CFAI Social domain | [social loneliness]*50/12 + [social support network]*50/10 |
| CFAI Environmental domain | [actual housing/environment]*100/20 |

| Table C: Cut-offs CFAI (18) | | | |
| --- | --- | --- | --- |
|  | No/Low frail | Mild frail | High frail |
| CFAI | 0.00 thru 21.89 | 21.90 thru 38.79 | 38.80 thru 100.00 |
| Physical domain | 0.00 thru 24.99 | 25.00 thru 75.00 | 75.01 thru 100.00 |
| Psychological domain | 0.00 thru 19.99 | 20.00 thru 45.84 | 45.85 thru 100.00 |
| Social domain | 0.00 thru 37.49 | 37.50 thru 64.15 | 64.16 thru 100.00 |
| Environmental domain | 0.00 thru 4.99 | 5.00 thru 30.00 | 30.01 thru 100.00 |
