# Supplementary material for: Concordances and differences between a unidimensional and multidimensional assessment of frailty: a cross-sectional study
Source: BMC Geriatr. 2019 Dec 10;19:346. doi: 10.1186/s12877-019-1369-7 (PMC6902576; doi:10.1186/s12877-019-1369-7)
Supplement: Supplementary file 4 — Additional file 4: Table S1. Observed agreement between Fried Phenotype and CFAI. [file 12877_2019_1369_MOESM4_ESM.docx]

**Additional Table S1: Observed agreement between Fried Phenotype and CFAI**

| Table A: Number of participants with different levels of frailty according to the Fried Phenotype and CFAI total | | | | | | |
| --- | --- | --- | --- | --- | --- | --- |
|  |  |  | Fried Phenotype | | |  |
|  |  |  | No | Pre-frail | Frail | Total |
| CFAI | No/low frail   N | | **31** | 51 | 4 | 86 |
|  | Mild frail | N | 13 | **48** | 11 | 72 |
|  | High frail | N | 2 | 10 | **23** | 35 |
|  | Total | N | 46 | 109 | 38 | 193 |
|  | | | | | | |

| Table B: Number of participants with different levels of frailty according to the Fried Phenotype and CFAI Physical | | | | | | |
| --- | --- | --- | --- | --- | --- | --- |
|  |  |  | Fried Phenotype | | |  |
|  |  |  | No | Pre-frail | Frail | Total |
| CFAI Physical | No/low frail | N | **37** | 64 | 4 | 105 |
|  | Mild frail | N | 9 | **40** | 24 | 73 |
|  | High frail | N | 0 | 7 | **10** | 17 |
|  | Total | N | 46 | 111 | 38 | 195 |
|  | | | | | | |

| Table C: Number of participants with different levels of frailty according to the Fried Phenotype and CFAI Psychological | | | | | | |
| --- | --- | --- | --- | --- | --- | --- |
|  |  |  | Fried Phenotype | | |  |
|  |  |  | No | Pre-frail | Frail | Total |
| CFAI Psychological | No/low frail | N | **34** | 72 | 10 | 116 |
|  | Mild frail | N | 10 | **30** | 16 | 56 |
|  | High frail | N | 2 | 7 | **12** | 21 |
|  | Total | N | 46 | 109 | 38 | 193 |
|  | | | | | | |

| Table D: Number of participants with different levels of frailty according to the Fried Phenotype and CFAI Social | | | | | | |
| --- | --- | --- | --- | --- | --- | --- |
|  |  |  | Fried Phenotype | | |  |
|  |  |  | No | Pre-frail | Frail | Total |
| CFAI Social | No/low frail | N | **13** | 36 | 9 | 58 |
|  | Mild frail | N | 25 | **58** | 18 | 101 |
|  | High frail | N | 8 | 17 | **11** | 36 |
|  | Total | N | 46 | 111 | 38 | 195 |
|  | | | | | | |

| Table E: Number of participants with different levels of frailty according to the Fried Phenotype and CFAI Environmental | | | | | | | | | |  |
| --- | --- | --- | --- | --- | --- | --- | --- | --- | --- | --- |
|  | |  |  | | Fried Phenotype | | |  | |  |
|  | |  |  | | No | Pre-frail | Frail | Total | |  |
| CFAI Environmental | | No/low frail | N | | **24** | 42 | 12 | 78 | |  |
|  | | Mild frail | N | | 19 | **65** | 20 | 104 | |  |
|  | | High frail | N | | 3 | 4 | **6** | 13 | |  |
|  | | Total | N | | 46 | 111 | 38 | 195 | |  |
|  | | | | | | | | | |  |
| Table F: The percentage of participants frail according to the Fried Phenotype, the CFAI or both frailty measurements | | | | | | | | | | |
|  | Fried Phenotype | | | Fried Phenotype and CFAI | | | | | CFAI | |
| Frailty | 7.77% | | | 11.92% | | | | | 6.21% | |
| Physical domain | 14.36% | | | 5.13% | | | | | 3.59% | |
| Psychological domain | 13.47% | | | 6.22% | | | | | 4.66% | |
| Social domain | 13.85% | | | 5.64% | | | | | 12.82% | |
| Environmental domain | 16.41% | | | 3.08% | | | | | 3.59% | |
|  | | | | | | | | | | |
