# Supplementary material for: Concordances and differences between a unidimensional and multidimensional assessment of frailty: a cross-sectional study
Source: BMC Geriatr. 2019 Dec 10;19:346. doi: 10.1186/s12877-019-1369-7 (PMC6902576; doi:10.1186/s12877-019-1369-7)
Supplement: Supplementary file 5 — Additional file 5: Table S2. Physical activity. [file 12877_2019_1369_MOESM5_ESM.docx]

**Additional table S2: physical activity**

Since physical activity is a part of the Fried Phenotype, an additional analysis whereby the item physical activity was not included in the Fried Phenotype was done. The table below presents these results. If physical activity was not included 27 persons met three or four criteria of the Fried Phenotype. The ANOVA suggested that there is a significant difference between the three groups (p=0.01531) indicating that persons frail according to FP did less physical activity.

|  | Solely CFAI | CFAI and FP | Solely FP |
| --- | --- | --- | --- |
|  | High frail | (High-) frail | Frail |
|  | N=12 | N=17 | N=10 |
| Never | 2 | 13 | 6 |
| Rarely | 1 | 1 | 0 |
| monthly | 1 | 1 | 0 |
| weekly | 8 | 2 | 4 |

P<0.05
